# Trapped in freshwater: the internal anatomy of the entoproct *Loxosomatoides sirindhornae*

**DOI:** 10.1186/1742-9994-7-7

**Published:** 2010-02-04

**Authors:** Thomas Schwaha, Timothy S Wood, Andreas Wanninger

**Affiliations:** 1 Department of theoretical biology, Morphology Section, University of Vienna, Althanstraße 14, Vienna, AT-1090, Austria; 2 Department of Biological Sciences, Wright State University, 3640 Colonel Glenn Highway, Dayton, OH 45435, USA; 3 Research Group for Comparative Zoology, Department of Biology, University of Copenhagen, Universitetsparken 15, Copenhagen, DK-2100, Denmark

## Abstract

**Background:**

Entoprocta is a small phylum of tentacle-bearing spiralian lophotrochozoans that comprises mainly marine representatives, with only two known freshwater species. One of them, *Loxosomatoides sirindhornae *Wood, 2005 was only recently described, and detailed information on its morphology including adaptations to life in freshwater are unknown. We analyzed the internal anatomy of *L. sirindhornae *using serial semi-thin sections, 3D reconstruction, as well as immunocytochemistry and confocal laserscanning microscopy.

**Results:**

The nephridial system shows high complexity, strikingly similar to that of *Urnatella gracilis*, the only other known freshwater entoproct. It is composed of 105-120 large flame-bulb terminal organs that occur in the stalk and calyx. In the stalk they terminate in the epidermis, whereas efferent ducts in each terminal organ in the calyx lead to large, paired terminal ducts that fuse close to the central nervous system and open into the atrium by a nephridiopore. Compared to other stolonate entoprocts, *L. sirindhornae *shows a different stalk-calyx junction by possessing only a single, multicellular canopy instead of a stack of star cells. A sphincter muscle is situated below the diaphragm of the stalk. The remaining musculature is concentrated in the stalk, while the calyx musculature is sparsely developed. The central nervous system is dumbbell-shaped as in basal entoprocts.

**Conclusions:**

The nephridial system probably has mainly osmoregulatory function. Previous studies have shown that *L. sirindhornae *is unable to cope with higher salinities, suggesting that its adaptation to freshwater has reached such a high degree that it is unable to 'turn off' the nephridial system in higher salinities. The current data available show that the architecture of internal organ systems such as the musculature or the calyx-stalk junction hold more promising information for taxonomic and perhaps even evolutionary inferences in Entoprocta than external characters such as spination. Contrary to previous investigations, the longitudinal calyx musculature of the genus *Loxosomatoides *should not be classified as generally strong or conspicuous, since its extent and site of insertion differs between species.

## Background

Entoprocta or Kamptozoa is a small phylum of sessile, marine, filter-feeding animals which most likely cluster with other spiral cleaving taxa within the protostomian superclade Lophotrochozoa [1]. To date, approximately 180 described species belonging to four families are recognized [2,3]. The family Loxosomatidae is the most species rich and comprises only solitary forms, while the remaining families Loxokalypodidae, Pedicellinidae and Barentsiidae are colonial. Only two species within the phylum live in freshwater habitats, the widespread *Urnatella gracilis *(Barentsiidae) and the recently described *Loxosomatoides sirindhornae *Wood, 2005. This species was discovered in two rivers from central Thailand and forms stolonate colonies with segmented stalks [4]. Since the genus *Loxosomatoides *belongs to the Pedicellinidae [4,5], it must be assumed that entoprocts have invaded freshwater at least twice independently in the course of their evolution [4].

The morphology, ecology and reproduction of *Urnatella gracilis *has been described in considerable detail [6-11]. As in other invertebrate phyla, freshwater environments have led to specific adaptations of this species such as, for example, the nephridial system, which is composed of numerous large terminal organs found in the calyx and stalk. The terminal organs are followed by thin contorted tubules that in the stalk end directly in the epidermis and in the calyx lead into large terminal ducts on the lateral sides. The terminal ducts meet in the middle below the atrial floor where they exit by a pore [8]. Additional adaptations to freshwater in *U. gracilis *are formation of hibernacula [12], which are also found in *L. sirindhornae *[4]. More adaptations are to be expected and require the investigation of internal structures, particularly the nephridial system. In addition, species differences within the genus *Loxosomatoides *and the related genus *Myosoma *are few [5]. Accordingly, this study focuses on the anatomy of *L. sirindhornae *including possible further adaptations to freshwater as well as new morphological characters applicable to taxonomic inferences.

## Methods

### Animals

Specimens of *Loxosomatoides sirindhornae*, Wood 2005 were collected from ropes dangling into the river Mae Klong close to the city Kanchanaburi, Thailand in February 2009. Pieces of rope with *L. sirindhornae *were transferred to the laboratories of the Department of Environmental Sciences of the Kasetsart University in Bangkok, where the samples were relaxed with a 1% solution of MgCl_2 _[see e.g. [13]]. However, the tentacles remained retracted in most specimens.

### Fixation, sectioning and 3D reconstruction

Specimens were fixed in a 2% glutaraldehyde solution in 0.01 M sodium-cacodylate buffer with a pH of 7.4 for 1 hour at room temperature. Subsequently, they were rinsed three times in the buffer and removed from the rope with forceps and sharp needles. Samples were afterwards postfixed in 1% osmiumtetroxide in distilled water for 1 hour at room temperature. Dehydration was carried out using acidified dimethoxypropane before embedding in Agar Low Viscosity resin using acetone as intermediate. Serial sectioning of eight specimens was conducted as described by Ruthensteiner [13] on a Reichert Ultracut S microtome at a sectioning thickness of 1 μm. Sections were stained with toluidine blue. Digital images were captured with a Leica DMRXA microscope equipped with an Evolution MP digital camera or a Nikon Eclipse E800 microscope equipped with a Nikon DS5-U1 camera and edited in Adobe Photoshop CS 2 or 3 (Adobe, San Jose, CA, USA). One complete longitudinal section series was taken for reconstructing the whole animal, while parts of a frontal section series was used for a detailed reconstruction of the nervous system and surrounding tissues such as the nephridial system. For 3D reconstruction, the image stacks were converted to greyscale and reduced in size prior to the import into the visualisation software Amira 4.1 (Mercury Computer Systems, Chelmsford, MA, USA) [see [13] for details]). Alignment was conducted automatically and where necessary corrected manually prior to manual segmentation of different organ systems. A surface of the segmented structure was created and optimised by iterated steps of simplification which was followed by smoothing. Snapshots of the reconstructions were taken with the Amira software.

### Immunocytochemistry and confocal microscopy

Specimens were fixed in 4% paraformaldehyde in 0.01 M phosphate buffer (PBS) containing 0.01% NaN_3 _for 1 hour at room temperature and thereafter rinsed three times in PBS. Samples were stored at 4°C in this solution until further processing. After three additional washes in PBS, the samples were permeabilized in PBS containing 4% Triton-X (PBT) for approximately 1 hour prior to staining for F-actin. This was followed by overnight incubation at 4°C in a 1:40 dilution of Alexa Fluor 488 phalloidin (Invitrogen, Molecular Probes, Eugene, OR, USA) in PBT. Specimens were then rinsed several times in PBS and mounted in Fluoromount G (Southern Biotech, Birmingham, AL, USA) on standard microscope slides. For alpha-tubulin staining, unspecific binding sites were first blocked in 6% normal goat serum (NGS) in PBT (block-PBT) overnight at 4°C. A mouse anti-acetylated alpha-tubulin antibody (Sigma, Brøndby, Denmark) was applied at a concentration of 1:300 in block-PBT for 24 hours. After several washes in block-PBT for 6 hours, an AlexaFluor 488 conjugated goat-anti mouse secondary antibody (Invitrogen, Molecular Probes, Eugene, OR, USA) was applied at a concentration of 1:200 (diluted in block-PBT) for 24 hours. Then, the samples were rinsed several times in PBS for about 6 hours before embedding in Fluoromount G.

Analysis and image acquisition was performed on a Leica DM IRBE microscope (Leica Microsystems, Wetzlar, Germany) equipped with a Leica TCS SP2 confocal unit. Confocal image stacks were scanned in steps of 0.5 to 1 μm along the Z-axis. Maximum intensity projections were generated with the supplied Leica software.

## Results

### General morphology

**Figure 1:**
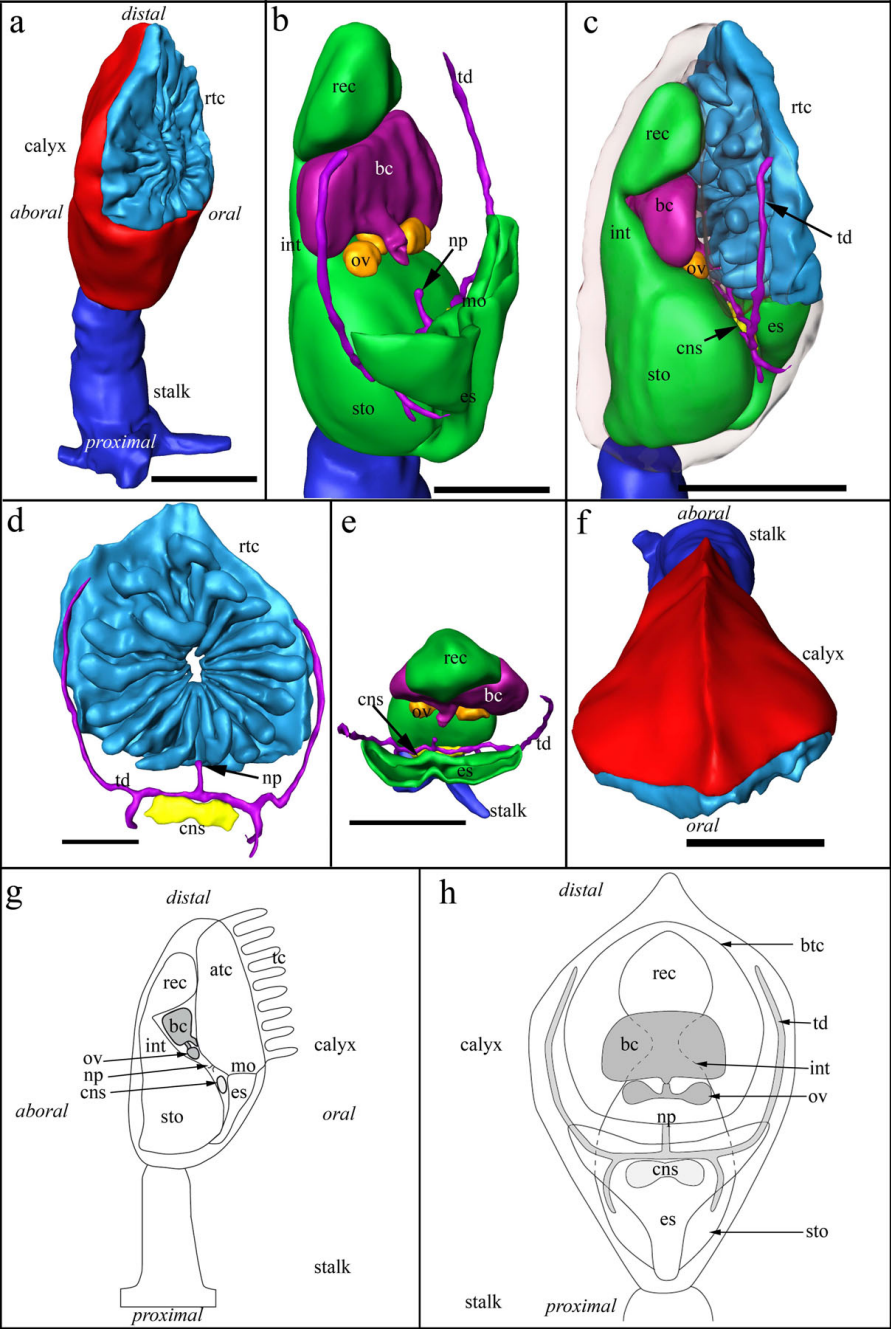
**3D reconstruction and schematic representations of *Loxosomatoides sirindhornae *based on serial sections**. (a) Overview showing the general shape of the animal divided into the stalk and calyx. (b) Oblique view into the internal anatomy, showing the U-shaped digestive tract, reproductive system including gonads and brood chamber and the terminal ducts of the nephridial system. (c) Lateral view; body of the calyx displayed transparently. (d) View on the retracted tentacle crown showing 17 tentacles, the terminal ducts of the protonephridial system and the ganglion. (e) Top view, tentacle crown and body of the calyx omitted. (f) Top view showing the aboral median carina on the calyx. Scale bar: 100 μm. (g) Schematic representation of the internal anatomy of *L. sirindhornae *in lateral view. Nephridial system with exception of the nephridiopore omitted. (h) Schematic representation of the internal anatomy of *L. sirindhornae *in frontal view. Tentacles are omitted for clarity. Colors: dark blue - stalk, red - body of the calyx, light blue - retracted tentacle crown, green - digestive tract, dark purple - terminal ducts of the nephridial system, light purple - brood chamber, orange - ovary + oviduct, yellow - central nervous system. Abbreviations: atc - atrial cavity, bc - brood chamber, btc - border of the tentacle crown, cns - central nervous system, es - esophagus, int - intestine, mo - mouth opening, np - nephridiopore, ov - ovary, rec - rectum rtc - retracted tentacle crown, sto - stomach, tc - tentacle crown, td - terminal duct.

**Figure 2:**
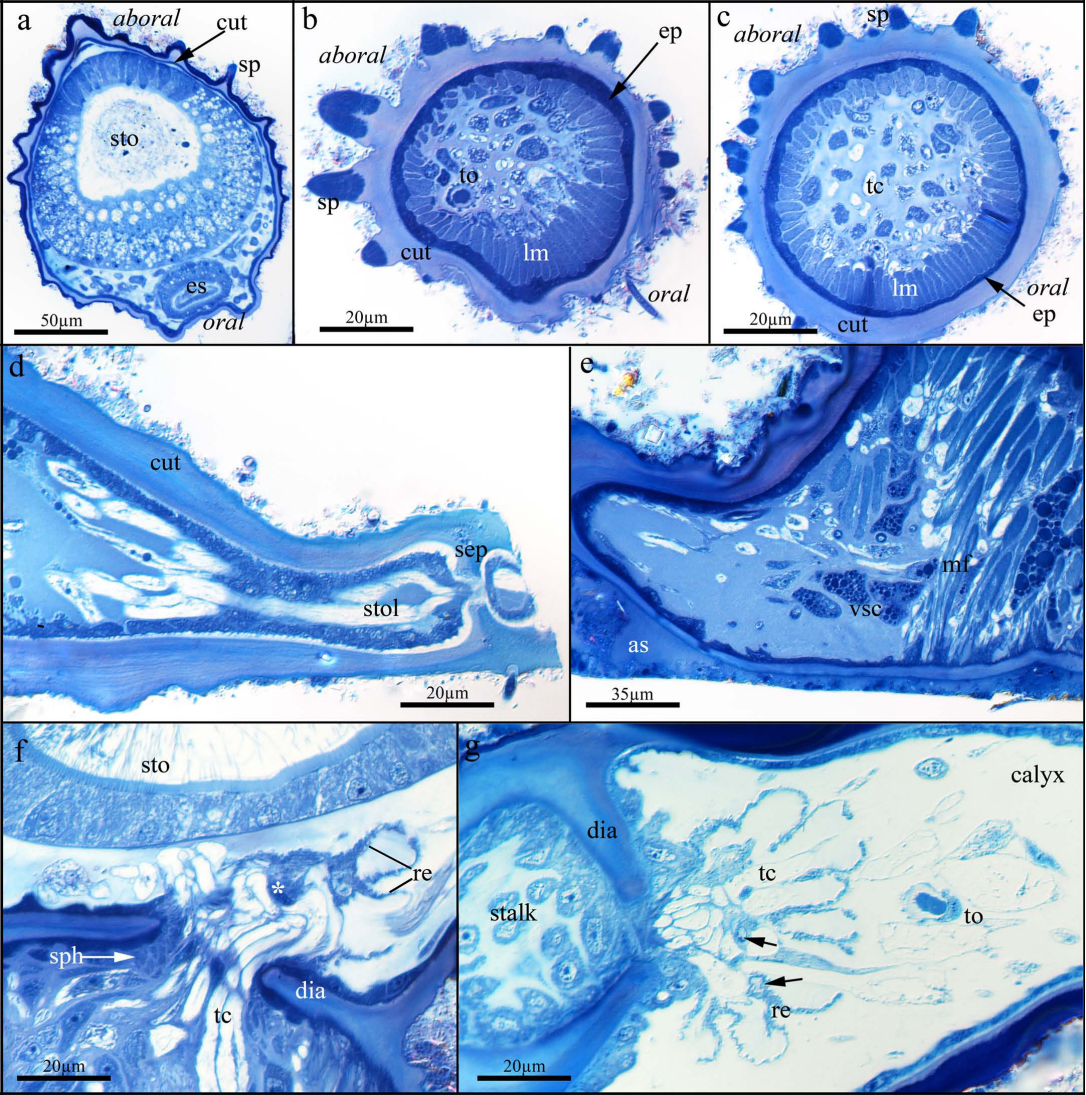
**Details of the stalk and cuticle of *Loxosomatoides sirindhornae***. (a) Cross section of the calyx showing the two layered cuticle concentrated on the aboral and lateral sides along with spines protruding from it. (b) Cross-section of the stalk of a juvenile specimen showing proportionally large spines. Also visible is a terminal organ inside the stalk. (c) Cross-section of the stalk of an adult specimen showing the rather thick cuticle with small spines. (d) Longitudinal section of a stolon showing a septum on the right side. The specimen has been cut on the right side, just after the septum. Note also the lamellar structure of the cuticle. (e) Base of the stalk showing muscle fibres attaching in the cuticle above the attachment site. Several mesenchymatous cells with prominently stained vesicles are visible. (f) Longitudinal section at the calyx-stalk junction, showing the cuticle diaphragm constricting the passage where several tubular cells extend from the stalk into the calyx. Bordering the median side of the diaphragm, several fibres of the stalk sphincter are visible. Above the diaphragm a multicellular 'star structure' (asterisk), shaped like a canopy, is visible. (g) Slightly oblique section through the stalk-calyx junction showing the 'star structure' with several cells (arrows) and radial extensions emanating from it where the tubular cells of the stalk pass through. Also note a terminal organ on the right side of the calyx. Abbreviations: as - attachment site, cut - cuticle, dia - diaphragm, ep - epidermis, es - esophagus, lm - longitudinal musculature, re - radial extensions, sep - septum, sp - spine, sph - sphincter, sto - stomach, stol - stolon, tc - tubular cell, to - terminal organ, vsc - stalk cells containing prominently staining vesicles.

The body of *L. sirindhornae *is divided into a muscular stalk and an obliquely oriented calyx (Fig. 1a, g). The stalk is circular in cross section along most of its length but flattens towards the substrate, to which it attaches by a homogenous, probably cuticular, pad (Figs.1a, b, c, e). Thin, creeping stolons are connected to the base. Septa with a central opening are distinguishable in the stolon (Fig. 2d). The calyx bears most of the animal's organs (Fig. 1b, c, e, g, h) and is of almost triangular shape in cross-section. Aborally, the calyx has a cuticular shield that is ornamented by a median carina (Fig. 1f). On the distal oral side it carries the tentacle crown with 16-17 tentacles. In their retracted condition, the tentacles are withdrawn into the atrium while the enclosed tentacular membrane has a wrinkled appearance leaving only a small orifice to the exterior (Fig. 1a, c, d).

The outer surface is covered by a cuticle that is thicker and two-layered at the lateral and aboral side of the calyx, as seen in its staining properties on sections (Fig. 2a). Several protuberances or small spines cover the outer cuticle (Fig.2a-c). These are concentrated on the aboral side and are proportionally larger in smaller specimens than in fully grown individuals (Fig. 2b, c). They show staining properties similar to the thickened aboral shield of the calyx. The remaining cuticle of the pedicle shows several rows of lamellae (Fig. 2b-e). A cuticular diaphragm constricts the connection between stalk and calyx. Centrally, several membranous tubules penetrate the opening and spread radially into the calyx. A distinct star-cell complex is not present; instead, a canopy-shaped structure composed of multiple cells is situated at the calyx-stalk junction. It bears several thin radial extensions where the tubular extensions from the stalk transit (Fig. 2f, g).

**Figure 3:**
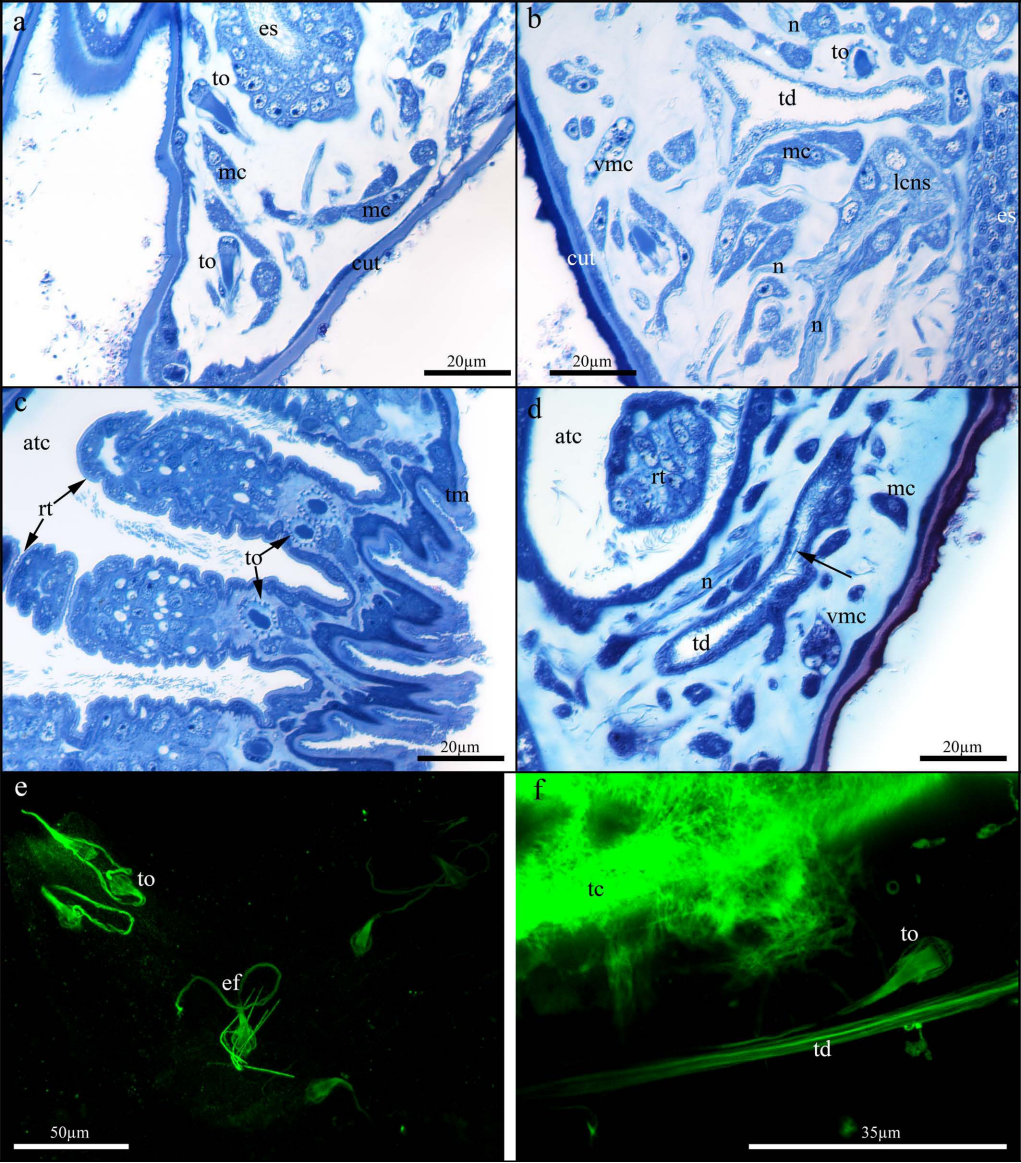
**Details of the nephridial system of *Loxosomatoides sirindhornae***. (a) Longitudinal section through the calyx showing flame bulbs close to the esophagus. (b). Longitudinal section slightly past the esophagus showing the large terminal duct and a lateral part of the central nervous system with nerves emanating from it. (c). Longitudinal section through the tentacle membrane with retracted tentacles. (d). Longitudinal section behind esophagus showing ciliation (arrow) in the terminal duct of the nephridial system. (e). Maximum intensity projection of a confocal image stack of alpha-tubulin staining in the stalk showing terminal organs and efferent ducts of the protonephridia. (f). Maximum intensity projection of a confocal image stack of alpha-tubulin staining showing a terminal organ and terminal duct of the protonephridial system in the calyx. Abbreviations: atc - atrial cavity, cut - cuticle, ef - efferent duct of the terminal organ, es - esophagus, lcns - lateral central nervous system, mc - mesenchymatous cell, n - nerve, rt - retracted tentacle, tc - tentacle cilia, td - terminal duct, tm - tentacle membrane, to - terminal organ, vmc - vesicular mesenchymatous cell.

The stalk itself is composed of the aforementioned cuticle with spines, followed by a distinct epidermal layer (Fig. 2b-e). Underneath, a complete ring of longitudinal muscle bundles traverse the stalk. Centrally, the stalk is filled by mesenchyme cells surrounded by homogenously staining extracellular matrix. Most of these cells are tubular cells that run in the longitudinal direction of the stalk (Fig. 2b, c). Additionally, several flame-bulb protonephridia (15-20 in adult specimens) are present in the stalk (Fig. 2b). Efferent ducts for each terminal organ are contorted and run to the epidermis of the stalk (Fig. 3e). At the proximal pole, close to the attachment base, the stalk contains multiple mesenchymatous cells that exhibit several prominently staining vesicles (Fig. 2e).

**Figure 4:**
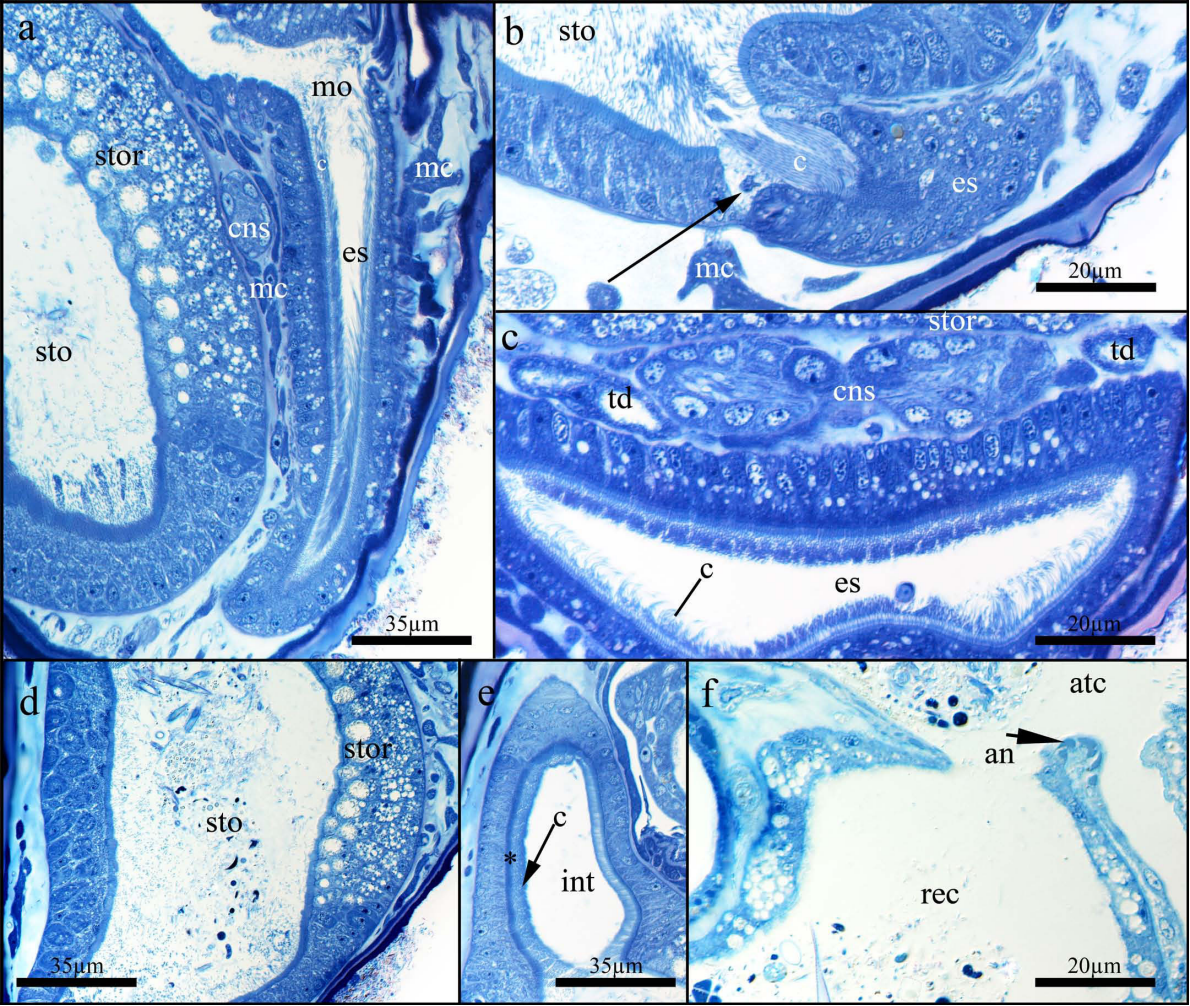
**Details of the digestive tract of *Loxosomatoides sirindhornae***. (a) Longitudinal section showing the ciliated mouth opening and esophagus on the right side as well as in parts of the stomach, including the conspicuous stomach roof on the left. The central nervous system and several mesenchymatous cells of the body cavity lie between the stomach and esophagus. (b) Detail of the esophagus-stomach transition showing the conspicuous cells at the border and the dense ciliation reaching into the stomach. (c) Cross-section through the esophagus showing its thicker and more vesicular side towards the stomach. The dumbbell-shape of the central nervous system is visible together with both terminal ducts of the nephridial system. (d) Longitudinal section showing details of the stomach. The orally situated roof of the stomach bears a conspicuous epithelium with many vesicles. (e) Longitudinal section through the intestine showing a prominently stained layer (asterisk) below the dense ciliation. (f) Details of the rectum with its conspicuous vesicular epithelium. Bordering the anal opening, several fibres of the anal sphincter are visible (arrow). Abbreviations: an - anus, atc - atrial cavity, c - cilia, cns - central nervous system, es - esophagus, int - intestine, mc - mesenchymatous cell, mo - mouth opening, rec - rectum, sto - stomach, stor - stomach roof, td - terminal duct.

**Figure 5:**
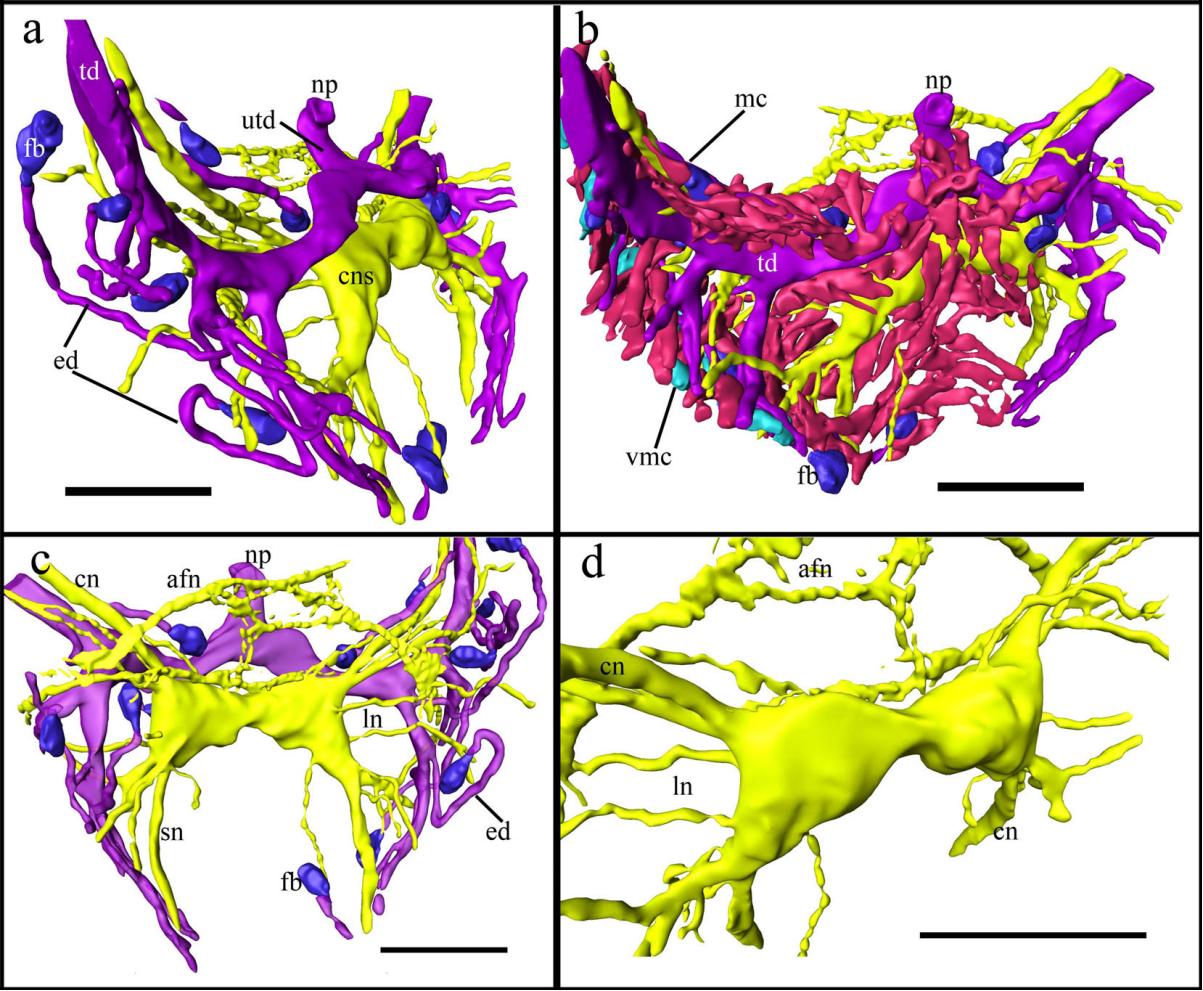
**3D reconstruction of the central nervous system of *Loxosomatoides sirindhornae *with peripheral nerves, part of the nephridial system and mesenchymatous cells of the body cavity**. Only a small part was reconstructed to demonstrate the complexity of these organ systems. (a) oblique-frontal view without mesenchymatous cells displayed. Note both terminal ducts joining above the central nervous system and opening by the single nephridiopore. (b) View from more frontally showing the complex web of mesenchymatous cells entangling the nervous and nephridial system. Close to the edge a couple of vesicular mesenchymatous cells are shown. (c) Aboral view of the nervous and nephridial system showing nerve trunks emanating from the central nervous system. (d) Close-up of the central nervous system and main nerves. Colors: Yellow - nervous system, purple - nephridial system, blue - flame bulbs/terminal organs, red - mesenchymatous cells, turquoise - vesicular mesenchymatous cells. Abbreviations: afn - atrial floor nerves, cn - nerve to calyx, cns - central nervous system, ed - efferent ducts of the nephridial system, fb - flame bulbs/terminal organs, ln - nerves innervating the lateral sides, mc - mesenchymatous cell, np - nephridiopore, sn - nerves to stalk, td - terminal duct, utd - unpaired terminal duct, vmc - vesicular mesenchymatous cell.

Apart from nerves and parts of the nephridial system, which will be described below, the body cavity of the calyx is filled with numerous polygonal, sometimes elongated cells. Several thin cytoplasmatic protrusions extend from each cell and interconnect the cells or attach to the bodywall and other organ systems such as the digestive tract or the nephridial system (Figs. 3a, b, d; 4a, b; 5b). Close to the body wall some of these cells exhibit a different appearance by possessing several vesicles (Figs. 3b, d; 5b).

### Digestive tract

The U-shaped digestive tract is composed of a mouth opening, esophagus, stomach, intestine and rectum (Fig. 1b, c, e, g, h). The mouth opening is laterally elongated and leads into the esophagus, which is flattened along the oral-aboral axis, while extending laterally on the distal side (Figs. 1e; 4c). At its beginning it is shaped like a flat funnel that subsequently narrows until it is circular in cross-section, before entering proximally into the stomach. The epithelium of the esophagus is densely ciliated with multiciliated cells and contains several vesicles (Fig. 4a, c). On the aboral side it is distinctly larger in its height than on the oral side. At the entrance to the stomach, a bundle of long cilia of the esophagus reaches into the stomach. Conspicuous triangular, pale cells border the entrance (Fig. 4b). The stomach is a large voluminous sac that fills almost half of the calyx (Fig. 1b, c, g). Its epithelium is covered by a prominently stained layer, perhaps microvilli. Proximally and along its median, aboral side it carries several cilia and has flat, polygonal to globular cells (Fig. 4a, d). The lateral sides and the orally facing roof of the stomach is composed of highly vacuolated cells forming a high columnar epithelium. The vesicles are filled with a granular substance. Basally, they are smaller and numerous, while apically, close to the stomach lumen, a single large vesicle is present (Fig. 4a, c, d). Distally, the stomach leads into a short intestine (Fig. 1b, c, g). The intestine is composed of regular cells without any conspicuous inclusions or vesicles (Fig. 4e). As in the stomach, these cells are covered by a prominently stained border. In addition, a regular, uniform ciliation is present. The rectum is ellipsoid in longitudinal aspect and almost triangular in cross-sectional aspect. It bears a thin epithelium with conspicuous vesicles. Its lumen is filled with debris and faeces and is closed by ring musculature (sphincter) towards the intestine. The rectum opens by the anus into the atrium (Fig. 4f).

### Nervous system

Between the stomach and the esophagus, slightly below the mouth opening, paired ganglia are present (Figs. 1c, d, h; 4a). Medially, they are connected by a commissure which gives the central nervous system (CNS) a dumbbell-like shape (Figs. 1h; 4c; 5c, d). Perikarya are located in the periphery, while the inner mass is composed of neuropil. Laterally, several nerve trunks originate, with the largest projecting distally into the lateral sides of the tentacle crown and proximo-frontally around the esophagus towards the stalk (Figs. 3b; 5a, c, d). At least two larger nerves originate in the middle of the lateral sides of the CNS. A number of smaller nerves that channel into a single bundle arch over the CNS, innervating the proximal atrial floor. Additional nerves extend from the area of the commissure into this arch (Fig. 5c, d).

### Nephridial system of the calyx

The protonephridial system is probably the most complex organ system in *L. sirindhornae*. In adult specimens, approximately 90-100 multi-ciliated, bulbous terminal organs with two nuclei are found in the calyx and in the stalk (Figs. 2b; 3a-c, e, f). In the calyx, terminal organs are concentrated at the oral side. One or two terminal organs lie at the base of each tentacle (Fig. 3c), while the remaining terminal organs are located around the esophagus. A few terminal organs are situated at the base of the calyx, close to the connection to the stalk (Fig. 2g) and aborally of the atrial wall. Each terminal organ leads to a thin, slightly convoluted capillary duct (Figs. 3f; 5a, c) that ends in a broad terminal duct. The terminal duct consists of two main lateral branches, each of which starting almost at the distalmost end next to the atrium (Figs. 1b-e, h; 3f). They continue proximally on the lateral sides until the height of the CNS, where they bend medially and then distally to unite into a single short duct that opens into the atrium (Figs. 1d, e, h; 3b; 4c; 5a-c). Two short branches split off where the main branches run medially and continue proximally on the lateral sides of the esophagus. At least parts of the terminal ducts seem to possess long cilia (Fig. 3d, f).

### Reproductive system

**Figure 6:**
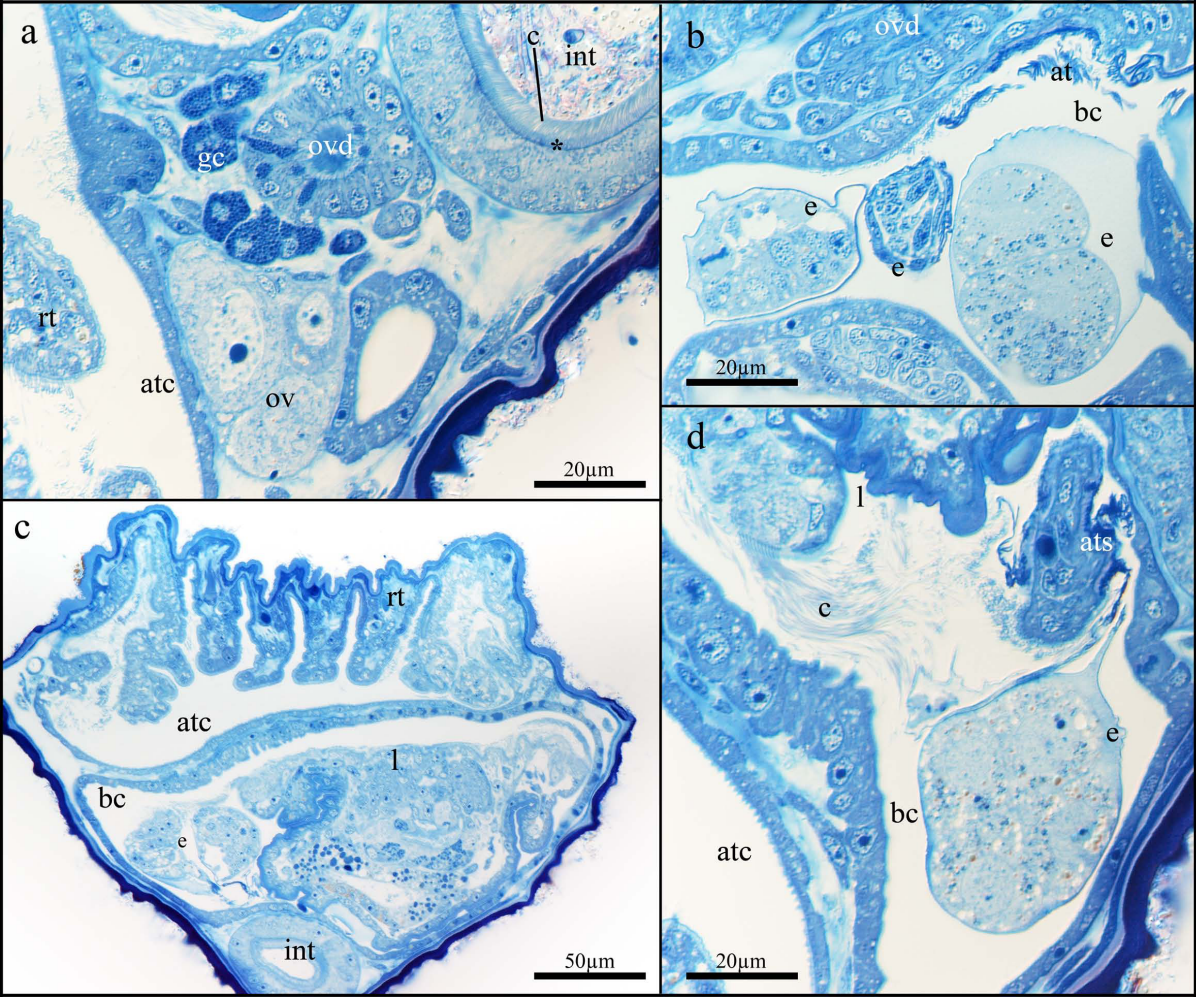
**Details of the reproductive system of *Loxosomatoides sirindhornae***. (a) Cross-section at the height of the intestine showing the prominently stained layer (asterisk) below the dense ciliation of the intestine as well as the ovary and oviduct with associated glands. (b) Detail of the brood chamber showing three embryos in different developmental stages. A two-cell stage is visible at the left side. At the upper right side several threads attaching embryos to the brood chamber are visible. (c) Cross-section of the calyx showing the large dimensions of the brood chamber with fully developed larvae. (d) Detail of the attachment site of the embryos within the brood chamber. Abbreviations: at - attachment threads, atc - atrial cavity, ats - attachment site of the embryos at the brood chamber, bc - brood chamber, c - cilia, e - embryo, gc - glandular cells, int - intestine, l - larva, ov - ovary, ovd - oviduct, rt - retracted tentacles.

The ovaries of *L. sirindhornae *are paired and lie laterally, slightly above the ganglion (Fig. 1b, c, e, g, h). Several large oocytes occur in each ovary (Fig. 6a). Two short ducts diverge from each ovary towards the sagittal plane, where they fuse into an unpaired oviduct that leads distally into a brooding pouch that lies next to the intestine and usually below the rectum (Figs. 1b, g; 6a). Close to the unpaired oviduct glandular cells are present that penetrate the epithelium of the duct and reach into its lumen (Fig. 6a). Up to six different developmental stages that are protected inside the brood pouch were found in a single animal, including two-and four-cell stages, early gastrulae as well as early and late larval stages (Fig. 6b-d). In four of the analysed specimens, however, four embryos were present in the brood pouch. Two other specimens contained two embryos and six embryos, respectively. A single specimen contained an early zygote, while the analysed medium-sized animal showed no sign of gonads or brood pouch. Early embryos are covered by a thin acellular envelope that is connected to the brooding pouch, close to the entrance of the unpaired oviduct, by thin threads emerging from the envelope (Fig. 6b, d). The envelope is ruptured once embryos have reached a certain size, but its remnants and connection to the brood pouch remain present.

The size of the brood pouch largely depends on the size of the embryos. With smaller embryos, the pouch is quite small and has a separate duct that traverses proximo-frontally and terminates at the level of the gonads with an opening close to the nephridial pore (Fig. 1b, d, g). When fully developed, larvae are present and, depending on their position, the brood pouch can extend almost to the lateral sides of the mother animal (Fig. 6c). Additionally, large and expanded brood pouches have been found where no separate duct to the atrium was present. We did not find male gonads in any of the specimens investigated.

### Myoanatomy

**Figure 7:**
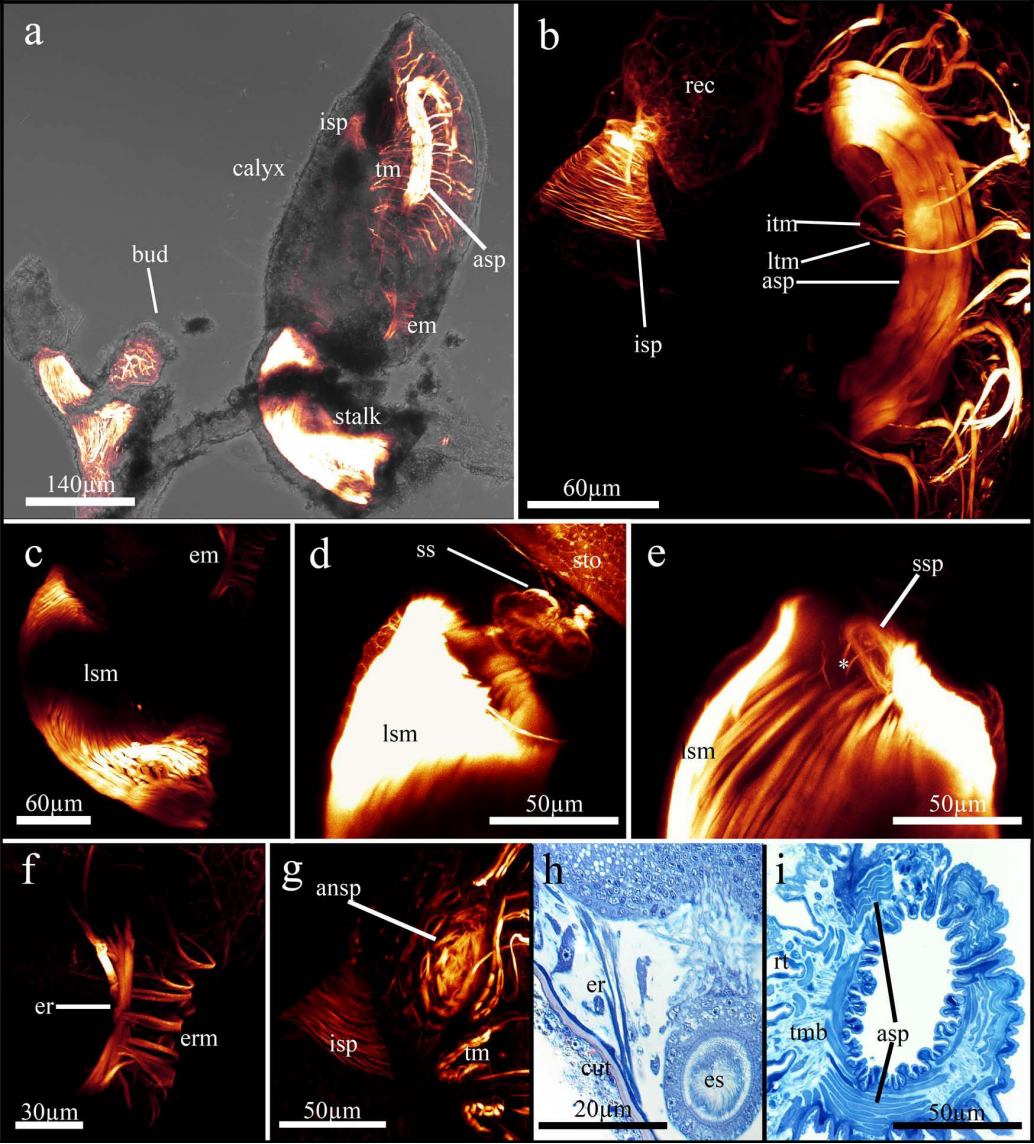
**Myoanatomy of *Loxosomatoides sirindhornae***. (a). General overview showing an adult on the right side with its muscular stalk and less muscular calyx. On the left side a small bud and a stalk without calyx are seen. (b) Lateral view of the tentacle crown with tentacle muscles and the atrial sphincter on the right. The intestinal sphincter is shown on the left side below the rectum. (c) Detail of the longitudinal muscles of the stalk, which is slightly bent in the specimen. At the right upper border the esophageal muscles are visible. (d) Detail of the 'star structure' in the calyx just below the stomach, somewhat above the end of the stalk. (e) Detail of the sphincter at the cuticular diaphragm of the stalk. A few small muscle fibres (asterisk) run from the sphincter towards the lateral musculature of the stalk. (f) Detail of the esophageal musculature showing conspicuous ring musculature and a few longitudinal muscles attaching to the upper end of the pharynx. (g) Detail of the anal sphincter opening into the atrium with retracted tentacles visible. Somewhat below, the intestinal sphincter is shown. (h) Frontal section of the calyx showing the esophageal retractors originating at the proximal bodywall and inserting at the upper broad end of the eosphagus. (i) Frontal section through the tentacle membrane and the contracted atrial sphincter. Abbreviations: ansp - anal sphincter, asp - atrial sphincter, cut - cuticle, em - esophageal musculature, er - esophageal retractors, erm - esophageal ring musculature, es - esophagus, isp - intestinal sphincter, itm - inner tentacle muscle, lsm - longitudinal stalk musculature, ltm - lateral tentacle muscle, rec - rectum, rt - retracted tentacles, ss -'star structure', ssp - stalk sphincter, sto - stomach, tm - tentacle musculature, tmb - tentacle membrane.

The musculature of *L. sirindhornae *is largely concentrated in the muscular stalk, where mainly continuous longitudinal musculature is present. At the aboral side it is somewhat thinner than on the oral side (Figs. 2b, c; 7a, c-e). It inserts proximally at the cuticle and distally at the septum that separates the stalk from the calyx. Below the diaphragm at the stalk-calyx junction, a sphincter is distinguishable (Figs. 2f; 7e). A few short muscle fibres extend from the sphincter into the lateral sides of the stalk (Fig. 7e). The canopy-shaped 'star-structure' contains several myofibrils (Fig. 7d).

In the calyx only few muscle groups are present. Most prominent is the ring musculature of the soft tentacular membrane on the oral side, that acts as a sphincter for enclosing the atrium (Fig. 7a, b, i). Each tentacle possesses two paired muscles, conspicuous main lateral tentacle muscles and very thin inner tentacle muscles (Fig. 7b). The digestive tract only shows ring musculature at the lower part of the esophagus and at the intestine, as well as an anal sphincter (Figs. 4f; 7a, b, f, g). The last group of calyx muscles are a few longitudinal muscle fibres that originate at the proximal bodywall and attach to the lateral sides of the esophagus (esophageal retractors) (Fig. 7f, h).

## Discussion

### Morphology of *Loxosomatoides sirindhornae*: additions to its first description

The general morphology of *Loxosomatoides sirindhornae *resembles that of other pedicellinids showing a muscular, unsegmented stalk and a calyx. In *L. sirindhornae *the calyx is obliquely oriented with the tentacle crown tilted orally as also seen in several congeners [5]. Such an orientation is found in most loxosomatids, but the barentsiid *Urnatella gracilis *likewise bears a very similar shape of the calyx [12]. The digestive tract consists of a wide mouth opening followed by the esophagus, a voluminous stomach, an intestine and a rectum. Thus, the general composition and also the structural details (i.e. ciliation, appearance of the epithelia) are more or less identical to other entoprocts [14].

The morphology of mesenchyme cells in the body cavity has been described as amoeboid for *Pedicellina cernua *[15], which was subsequently adopted for all entoprocts in most compendia on the phylum [16,17]. However, their existence was subsequently rejected by Emschermann [18]. Also in *L. sirindhornae *these cells are not amoeboid, but merely possess several spinuous cytoplasmic connections to other cells and organ systems. Since they are most abundant around the esophagus, they appear to act as a stabilizing unit that ensures all organs are kept in place when the animal contracts the esophageal retractors. Adding to the original description [4], it should be noted that a diaphragm with a primitive 'star-structure' is present in *L. sirindhornae*. Septa within stolons have previously only been detected at hibernacula in *L. sirindhornae*, whereas they also regularly occur between individual zooids in the stolon.

### Structure of the calyx-stalk junction in colonial entoprocts

**Figure 8:**
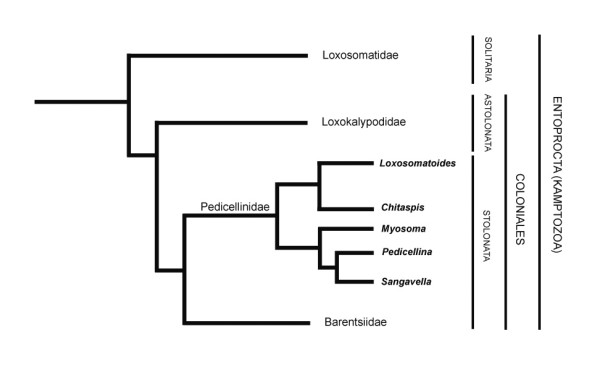
**Phylogenetic relationships of recent entoproct families, modified after Emschermann 1972**.

Mainly because of their creeping-type larva, solitary entoprocts belonging to the family Loxosomatidae are regarded as the most ancestral forms [19]. The stalk in loxosomatids is unconstricted as in the monotypic Loxokalypodidae. *Loxokalypus socialis *represents an intermediate form by possessing a supposedly plesiomorphic unconstricted stalk with continuous longitudinal stalk-calyx musculature. The formation of colonies unites this species with the remaining colonial families Pedicellinidae and Barentsiidae (Stolonata), which are characterized by a different colony form where individual zooids are connected by stolons (Fig. 8). A further apomorphy in the Stolonata is the strong cuticularisation of the stalk and the strong separation of stalk and calyx often exemplified by a cuticular diaphragm. While stolonate entoprocts usually exhibit a so-called star-cell complex, composed of a stack of flat, star-shaped cells in the stalk-calyx junction [20], *L. sirindhornae *shows only a single multi-celled canopy. This canopy is also present in other stolonate entoprocts, but in that case composed of a single cell - a so called cap-cell [21]. Either the multi-celled canopy structure of *L. sirindhornae *is derived from the stack of star cells or it represents an ancestral state in the evolution of the entoproct star cell complex. A similar condition as in *L. sirindhornae *seems to be present in *L. colonialis*, where only a single layer of flattened cells has been described [22]. This single layer of cells in *L. colonialis *has been previously interpreted as representing an early step in the evolution of the star-cell complex. Based on this differentiation of the star-cell complex and the shape of the longitudinal calyx musculature (see below), *Loxosomatoides *and related genera have been regarded as an early offshoot within stolonate entoprocts and thus as sister group to the remaining species [2]. However, for *L. laevis *an elaborate star-cell complex of five to eight cells has been described [5]. The closely related *Myosoma spinosa *also exhibits a distinct star-cell complex [23,24], whereas no detailed information exists for *M. hancocki *[25] and *Chitaspis athleticus *[20,26], respectively.

### Comparison of entoproct muscle systems

With the strict separation of calyx and stalk in most colonial entoprocts and the increased rigidity by a thick cuticle, several muscle systems have become dispensable. Therefore, the muscular system of *L. sirindhornae *is rather sparsely developed compared to muscular systems of the Loxosomatidae [27,28]. Most of the occurring muscles, however, are comparable to loxosomatids. Merely the sphincter below the stalk-calyx diaphragm represents a new muscle, which to our knowledge has not been reported from other colonial stolonates. It appears most probable that the sphincter interacts with the star-shaped structure as a circulatory organ, similar to colonial species with a star cell complex.

The tentacle musculature with outer main muscles and thinner inner muscles is identical to the analysed species of the Loxosomatidae [27-29]. There are two atrial ring muscles in both loxosomatids, but only a single thick muscle in *L. sirindhornae*. The muscles associated with the digestive tract (esophageal ring muscles, intestinal sphincter and anal sphincter) are similar to *Loxosomella *sp. and *Loxosoma *sp. [14,27,28]. Remaining muscles of the digestive tract as found in loxosomatids, i.e., rectal retractors and rectum musculature, are not present. The esophageal retractors found in *L. sirindhornae *are most probably derived from longitudinal stalk muscles that extend into the calyx as in loxosomatids [27,28]. With the formation of a cuticular diaphragm at the calyx-stalk junction, these muscles were separated from the stalk and gained new attachment sites. Functionally, they probably act as atrial depressors enlarging the atrial space during contraction, since the inflexible cuticle of the calyx is unable to give way. This longitudinal calyx musculature has been previously referred to as atrial retractors. Since these muscles are considered remnants of the continuous stalk-calyx musculature in loxosomatids [2], they are potentially important to clarify the internal relationships of entoproct taxa. Accordingly, *Loxosomatoides *and related genera are regarded as sister groups to the remaining pedicellinids by the presence of conspicuous and strong longitudinal calyx musculature as well as the structure of the calyx-stalk junction [5] (Fig. 8). The longitudinal calyx musculature is thought to have been progressively lost in more derived genera such as *Pedicellina *and *Barentsia *[2]. However, care should be taken with these assumptions especially since differences in the muscular system of the calyx and stalk seem to occur between the different species. In some of the species the calyx musculature is not as strong or conspicuous as previously assumed. Also, the point of their insertion seems to differ between species. In *L. sirindhornae *few and in *L. colonialis *multiple longitudinal muscles run orally from the base of the calyx to the esophagus [[5], this study]. For *Chitaspis athleticus*, Annandale [26] described two muscle strands in the oral part that traverse in the body wall. One of these muscles probably inserts at the stomach wall. Only for *L. laevis *strong oral calyx muscles have been described that insert at the atrial wall.

The presence of oblique stalk musculature has been used as taxonomic character for the genus *Myosoma*, but it also occurs in *Chitaspis athleticus*. The genus *Chitaspis *was characterised by the absence of stalk muscles on the aboral side [26]. In several species of *Loxosomatoides *(*L. colonialis*, *L. laevis*, *L. sirindhornae*) the oral stalk musculature is somewhat thicker than the aboral musculature [[5], this study]. Whether the aboral stalk musculature in *Chitaspis athleticus *is absent or only thinner as in loxosomatoids and whether or not this genus should be synonymised with *Loxosomatoides *as recently suggested [5] remains unresolved until thorough reinvestigations will be performed.

### The nephridial system in entoprocts and its significance in freshwater species

Adult entoprocts usually possess a single pair of protonephridia between the stomach and esophagus, that opens into the atrium by separate nephropores in loxosomatids or a single pore in pedicellinids and barentsiids [14,30]. By contrast, freshwater species such as *Urnatella gracilis *and *Loxosomatoides sirindhornae *show a complex branched nephridial system [[8], this study]. The systems of both species show striking similarities in the calyx and the stalk. In the calyx, both possess long terminal ducts that branch off small capillary ducts to numerous terminal organs. The terminal ducts are much longer and branched in *L. sirindhornae*, extending even into the lateral border of the tentacle crown. In *U. gracilis *they are restricted to the area between stomach and esophagus and are arranged in the shape of an inverted Y [8]. The protonephridia in the stalk show a similar structure as in the calyx, with bulbous terminal organs with contorted tubules. In both species they exit through the epidermis and in *U. gracilis *function in ion regulation [8]. Direct evidence for the function in *L. sirindhornae *is lacking, but most likely similar to *U. gracilis*. The two freshwater species are the only known species possessing protonephridia in the stalk. Many other colonial entoprocts have several pore organs with rudimentary cilia underneath the cuticle of the stalk which act as ion regulatory organs [31]. It has been assumed that they represent rudimentary protonephridia and that the ancestor of colonial entoprocts possibly had several protonephridia along the body surface [31]. Although *Loxosomatoides *appears to be a sister group to the remaining stolonate entoprocts, it seems rather unlikely to assume such an ancestor from the current state of knowledge. Unless similar conditions will be found in marine or brackish species of this genus, it seems more likely that protonephridia in the stalk are a special adaptation to freshwater.

Terminal organs in entoprocts are always flame-bulb nephridia composed of two cells and are usually small and thin [30]. In the freshwater species, however, they are large and bulbous, not to mention numerous [8]. As previously mentioned, the freshwater species currently belong to two different families and convergent adaptation to freshwater is most likely. It seems that a highly sophisticated nephridial system is a prerequisite for conquering freshwater in these species. Even developing trochophore-like larvae of *L. sirinhornae *possess four large terminal organs (Schwaha: unpublished observations), while all other known larvae, even that of *Urnatella gracilis*, have only one pair [7,14,32]. In *U. gracilis *the nephridial system appears to have solely osmoregulatory function. Furthermore, exposure to different salinities has shown that the terminal organs of *U. gracilis *decrease their beating frequencies in higher salinities and even stop beating at a certain concentration [8]. Albeit direct observation is missing, it is very likely that the protonephridial system functions similarly in *L. sirindhornae *and that, as in other entoprocts, all excretory processes take place in the stomach roof and rectum as indicated by their similar structure [8,33]. Similar to *U. gracilis*, *L. sirindhornae *is highly intolerant towards higher salinities [4]. Obviously, both freshwater entoprocts are hypertonic towards their surrounding medium and remove inflowing water with the protonephridial system. Despite that, both species are so well adapted to freshwater, that they are seemingly unable to properly 'turn off' this water-removal system. Unfortunately, concise information on the nephridial system of brackish species, e.g., of other loxosomatoids, is still lacking, but following our line of thought it is tempting to speculate that these species represent an intermediate type concerning the differentiation of the nephridial system.

### Anatomy of the central nervous system in entoprocts

The nervous system of entoprocts has only been the subject of very few detailed analyses. The dumbbell-like shape of the CNS, as for example found in representatives of the solitary Loxosomatidae, probably represents the plesiomorphic condition [27,34]. In some of the investigated species of the colonial Barentsiidae and Pedicellinidae an oval CNS has been described [15,35]. It has therefore been assumed that the oval ganglion results from a fusion of the paired ganglia [27]. *Loxosomatoides sirindhornae *still shows the plesiomorphic condition. Since the genus is considered as an early offshoot of stolonate species, this finding is congruent with the sparse solid entoproct phylogenies currently available [2]. However, the other freshwater species, *Urnatella gracilis*, likewise exhibits a dumbbell-shaped CNS [6], although it is currently recognized as a derived form of *Barentsia *[2,12]. Accordingly, the dumbbell-like shape would have re-evolved from the oval CNS of barentsiids. This scenario seems unlikely and it is more parsimonious to assign *U. gracilis *a more basal position within stolonate entoprocts, although phylogenetic analyses that corroborate such a scenario are currently lacking.

### Structure of the genital tract in entoprocts and reproductive aspects

The general organisation of the female genital tract resembles those of other entoprocts [14,15]. As in *L. sirindhornae*, maturation of few germinal cells in the ovary is common [33], but in *Urnatella gracilis *only a single oocyte matures at a time [7]. Glandular portions of the oviduct or 'shell glands' are common and produce egg envelopes and attachment cords [14,33]. While the shell gland is a compact large organ in *Loxosomella elegans *[14], it appears grape-shaped in *Loxosomatoides sirindhornae*, with several grouped cells that each lead into the oviduct by a thin cellular extension. As in most other species, direct evidence of fertilization is lacking, but can be assumed to take place in the ovary before the formation of the egg envelope [14].

By the absence of male gonads in all analysed specimens, it can be concluded that *L. sirindhornae *is a protandric hermaphrodite, similar to other members of the genus (*L. colonialis *and *L. evelinae*; see [22,36]). All adult specimens contained several embryos or larvae brooded inside the brooding pouch which occupies extensive space that might make further fertilization events unnecessary.

### Conclusions and outlook

Unlike other freshwater organisms (e.g. ectoprocts, bivalves and rotifers), the entoprocts show a drastically modified nephridial system. This probably explains why only two species have hitherto been found in freshwater. Previous studies of the *Loxosomatoides*-*Myosoma *species complex were mainly based on external features such as spines and ornamentation of the aboral shield [5]. Our study shows that the internal anatomy of these species such as the myoanatomy, structure of the stalk-calyx junction as well as the nephridial system holds more promising information for taxonomic and perhaps even evolutionary inferences. Thus, besides external features, more attention should be paid to sectioning methods and muscle staining analysed with confocal microscopy. In particular, features such as spines are troublesome as they might underlie ecological factors and therefore may vary greatly in size between individual zooids, as, e.g., seen in *L. colonialis *[5]. Also, with the exception of *L. evelinae*, all species of *Loxosomatoides *investigated so far possess polygonal ornamentation on the aboral shield, which is lacking in *L. sirindhornae*. Therefore, it should be questioned if the investigated species belongs to *Loxosomatoides *or not, especially since it also shows differences with respect to the, probably primitive, differentiation of the stalk-calyx junction. This junction, as well as differences in the musculature, seem to yield most information relevant for taxonomic analyses, but these characters are also important for understanding the evolution of colonialism in Entoprocta. Comparative data is unfortunately mostly missing or in definite need for revision for this neglected phylum, but hopefully will become available in the near future.

## Competing interests

The authors declare that they have no competing interesting.

## Authors' contributions

TS conducted all practical work and drafted the manuscript. TW coordinated research in Thailand, collected and identified the animals and contributed significantly to the manuscript. AW provided necessary facilities for providing research and contributed significantly to the writing of the manuscript. All authors read and approved the final version of the manuscript.
